# Osteopontin drives retinal ganglion cell resiliency in glaucomatous optic neuropathy

**DOI:** 10.1016/j.celrep.2023.113038

**Published:** 2023-08-23

**Authors:** Mengya Zhao, Kenichi Toma, Benyam Kinde, Liang Li, Amit K. Patel, Kong-Yan Wu, Matthew R. Lum, Chengxi Tan, Jody E. Hooper, Arnold R. Kriegstein, Anna La Torre, Yaping Joyce Liao, Derek S. Welsbie, Yang Hu, Ying Han, Xin Duan

**Affiliations:** 1 Department of Ophthalmology, University of California San Francisco, San Francisco, CA 94158, USA; 2 Department of Ophthalmology, Stanford University School of Medicine, Palo Alto, CA 94304, USA; 3 Viterbi Family Department of Ophthalmology, University of California San Diego, San Diego, CA 92037, USA; 4 Department of Pathology, Stanford University School of Medicine, Palo Alto, CA 94304, USA; 5 Department of Neurology and The Eli and Edythe Broad Center of Regeneration Medicine and Stem Cell Research, University of California San Francisco, San Francisco, CA 94143, USA; 6 Department of Cell Biology and Human Anatomy, University of California, Davis, Davis, CA 95616, USA; 7 These authors contributed equally; 8 Lead contact

## Abstract

Chronic neurodegeneration and acute injuries lead to neuron losses via diverse processes. We compared retinal ganglion cell (RGC) responses between chronic glaucomatous conditions and the acute injury model. Among major RGC subclasses, αRGCs and intrinsically photosensitive RGCs (ipRGCs) preferentially survive glaucomatous conditions, similar to findings in the retina subject to axotomy. Focusing on an αRGCs intrinsic factor, Osteopontin (secreted phosphoprotein 1 [Spp1]), we found an ectopic neuronal expression of Osteopontin (Spp1) in other RGCs subject to glaucomatous conditions. This contrasted with the Spp1 downregulation subject to axotomy. αRGC-specific Spp1 elimination led to significant αRGC loss, diminishing their resiliency. Spp1 overexpression led to robust neuroprotection of susceptible RGC subclasses under glaucomatous conditions. In contrast, Spp1 overexpression did not significantly protect RGCs subject to axotomy. Additionally, SPP1 marked adult human RGC subsets with large somata and SPP1 expression in the aqueous humor correlated with glaucoma severity. Our study reveals Spp1’s role in mediating neuronal resiliency in glaucoma.

## In brief

Zhao et al. reported that retinal neuron subpopulations are resilient with ectopic Spp1 expression, subject to an experimental glaucomatous model with ocular hypertension. Spp1 overexpression renders other susceptible retinal neurons with neuroprotection subject to glaucomatous conditions. Elevated SPP1 levels in aqueous humor may be biomarkers for human glaucoma progression.

## Graphical Abstract

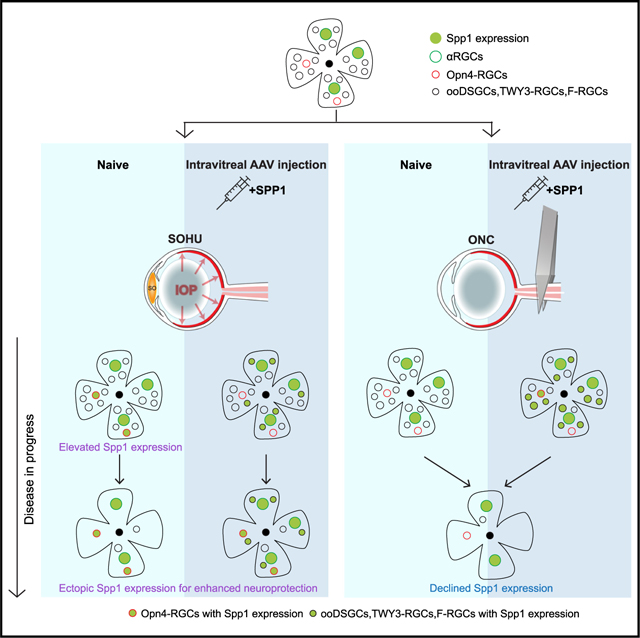

## INTRODUCTION

Irreversible neuronal loss under neurodegenerative conditions causes devastating functional changes to the mammalian central nervous system (CNS). Interestingly, neurodegeneration does not damage all neurons equally. At the cellular level, it has become apparent that cell death is not uniform, biasing certain neuron types.^1,2^ In the past, resilient and susceptible neurons were categorized based on gross anatomy or immunohistochemistry approaches. Recent genetics and single-cell transcriptomics advances have allowed a much higher-resolution look at these different populations. These efforts provided a comprehensive definition of neuronal types based on neuronal morphologies, functional properties, and molecular markers.^3^ These new technologies provide tools to monitor the pathological progression of various neurological injuries or degenerative conditions. Furthermore, beyond neuronal taxonomy, one can look into intrinsic molecular profiles of each neuronal resilient type for molecular insights against degeneration or injuries.^4^

The retina provides a perfect mammalian CNS model to understand how neurodegenerative conditions impact diverse neuronal types. We focused on optic neuropathies, the leading cause of irreversible blindness due to neurodegeneration.^5^ Optic neuropathies, including glaucoma, lead to the slow but relentless death of retinal ganglion cells (RGCs), the sole conduits of visual information from the eye to the brain. In mice, there are ~45 types of RGCs processing distinct visual features with distinct molecular signatures.^6^ Past studies, including ours, have utilized mouse genetics and single-cell transcriptomics methods to define neuronal responses of individual RGC subclasses or subtypes to acute injury.^6,7^ These experiments used the optic nerve crush (ONC) model to understand the mechanisms underlying acute axotomies.^8^ We demonstrated that RGCs are not uniformly susceptible to axotomy but rather have subclass-specific responses.^7^ Further, using genetically encoded RGC marking lines, we and others have shown that αRGCs and intrinsically photosensitive RGCs (ipRGCs) preferentially survive the ONC treatment.^7,9,10^ We also found that αRGCs had enriched and intrinsic Osteopontin (secreted phosphoprotein 1 [Spp1]) expression, with the potential for neuronal repair. We and others showed that Spp1 contributed to neuronal intrinsic axon regeneration in the retina and other CNS parts.^7,11,12^

We conducted a systematic RGC-type survey using the set of genetic lines and analyzed RGC cellular responses under chronic neurodegenerative conditions, modeling glaucoma. Surgery-based glaucoma models were established, leading to elevated intraocular pressure (IOP), such as the microbeads injection model.^13^ Past studies primarily utilized sparse genegun labeling methods or a few established transgenic RGC marking lines to measure individual RGC responses to IOP changes,^14,15^ including changes in dendritic morphologies and synaptic function changes at the individual neuron level. However, two factors have limited the progress of these experiments: (1) elevated IOPs are generally hard to control consistently over a prolonged time across many animals, and (2) these RGC marking lines were established for developmental studies and needed long-term marking in adults.^16,17^ To solve these issues, we recently established the silicone oil-induced ocular hypertension under-detected (SOHU) model for sustained IOP elevation, replicating the secondary glaucoma conditions in humans after silicone oil blocks the pupil.^18^ In parallel, we established a new collection of RGC Cre-based marking lines suitable for adult RGC subclass labeling.^19–22^

We applied the SOHU model to these Cre lines and examined whether different RGC subclasses exhibit preferential survival under glaucomatous conditions. We found that ocular hypertension leads to general RGC loss with preferential survival of αRGCs and ipRGCs. Spp1, an αRGC-enriched factor, was elevated in αRGCs and ectopically in ipRGCs subject to prolonged IOP elevation. This contrasted with the sharp decline of Spp1 under ONC conditions. Additionally, Spp1 offers these selective RGC subclasses (αRGCs and ipRGCs) distinct resiliency under SOHU-treated conditions. Genetic elimination of Spp1 reversed their preferential survival, suggesting that Spp1 is responsible for their resiliency. Conversely, Spp1 overexpression in the otherwise susceptible Foxp2-positive RGC subclass (F-RGCs) resulted in their enhanced survival under SOHU-treated conditions. Notably, Spp1-mediated neuroprotection was absent under ONC conditions. These data suggested Spp1’s role in chronic, but not acute, neuronal injury. Translating these findings to humans, SPP1 is enriched in RGCs with relatively large somata in adults but not in prenatal RGCs, as a marker for adult RGC subsets. Additionally, the secreted SPP1 concentrations in the aqueous humor of patients with glaucoma corresponded with disease severity. Our studies highlight how the intrinsic neuronal properties of RGCs affect their differential neuroprotective abilities subject to optic neuropathies. Molecules, such as SPP1, may serve as biomarkers for early diagnosis or be exploited to prevent vision loss in glaucomatous optic neuropathy.

## RESULTS

### Ocular hypertension leads to preferential survival of αRGCs and ipRGCs

**Figure 1.**
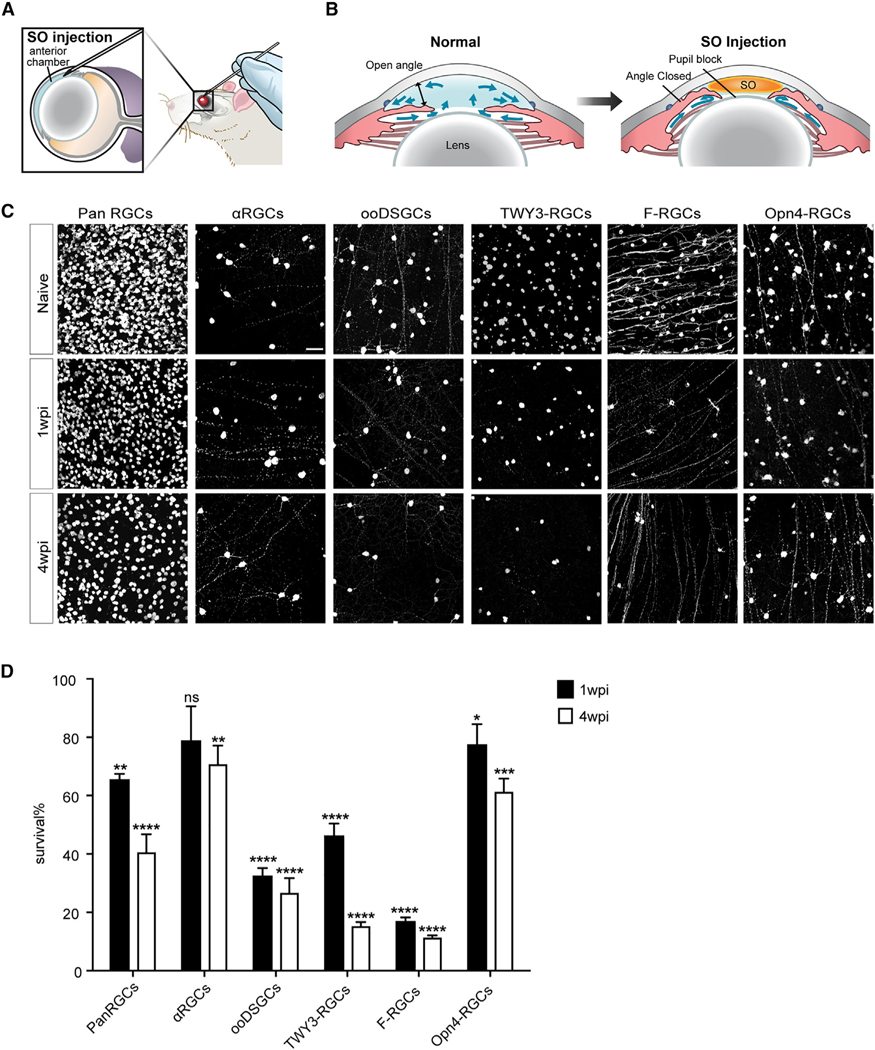
Ocular hypertension leads to preferential survival of αRGCs and ipRGCs (A and B) Illustration of the SOHU model surgery using the mouse eye (A) and the physical mechanisms resulting in elevated IOP after SOHU treatment (B). SO, silicone oil. (C) Whole-mount views of retinas among all RGCs and RGC subclasses. Retinas labeled ‘‘pan RGC’’ are labeled with the antibody RBPMS, which marks allRGCs. The rest of the retinas were from Kcng4-YFP, Cartpt-YFP, TWY3-YFP, Foxp2-YFP, and Opn4-YFP mice, in which αRGCs, ooDSGCs, W3-RGCs, F-RGCs, and Opn4-RGCs, respectively, are labeled genetically. Naive, sham-treated contralateral eye; wpi, weeks post-injection. Scale bar, 50 μm. (D) Fraction of pan RGCs and each subclass that survived SOHU treatment at 1 and 4 wpi. n = 5–8 animals per genotype. Data are presented as means ± SEM. The quantifications were generated by comparing 1 or 4 wpi with the naive baseline (100%). Unpaired two-sided Student’s t test, ns, not significant; ****p < 0.0001; ***p < 0.001; **p < 0.01; *p < 0.05.

We applied the SOHU model onto multiple RGC marking lines to examine the effects of ocular hypertension. Consistent with past work,^18^ the injection of a silicone oil drop into the anterior chamber of the mouse retina (Figures 1A and 1B) led to an elevated IOP over a prolonged window (Figure S1A) (24 ± 10 mmHg over 4 weeks of treatment, see STAR Methods for details). Thus, the SOHU model is highly suitable for testing the chronic effects of ocular hypertension over a large cohort of animals. We also compared the SOHU model with another well-established experimental glaucoma model, the microbead-induced ocular hypertension model.^13,23^ We showed that injecting microbeads into the anterior chamber resulted in a sustained increase in IOP (Figure S1A) in the range of 16 ± 1 mmHg, with a slightly lower IOP than the SOHU model. The microbead occlusion model also resulted in ~60% survival of RGCs by 4 weeks post-injection (wpi; Figures S1B and S1C). Collectively, our comparative data ensured that the SOHU model recapitulates the RGC loss seen in glaucoma due to chronic IOP changes. Compared with other experimental glaucoma models, the SOHU model offers prolonged regulation of the level and duration of the increase in IOP.

We collected Cre knockin lines that provide reliable adult RGC subclass labeling.^19^ Genetic crosses led to Cre-dependent GFP expressions as permanent markings of RGCs throughout their life. These contrast the first generation of RGC marking lines for developmental studies, which largely rely on BAC-transgenic labeling methods.^16,17^ The major RGC subclasses in the Cre knockin lines include αRGCs (Kcng4-YFP, 4 individual RGC subtypes), ON-OFF direction-selective ganglion cells (abbreviated as ooDSGCs; Cartpt-RGCs, 4 subtypes), W3-RGCs (TYW3-YFP, 5 subtypes), F-RGCs (Foxp2-RGCs, 4 subtypes), and ipRGCs (Opn4-YFP, 5 subtypes), covering about half of all RGC subtypes.^6^ Among these subclasses, we found that the αRGCs preferentially survived at 1 and 4 wpi relative to the ooDSGCs, W3-RGCs, F-RGCs, and the total RGC population (Rbpms positive) (Figures 1C and 1D). In addition, ipRGCs also demonstrated preferential survival at 1 and 4 wpi (Figures 1C and 1D). These data suggest that αRGCs, and to a lesser extent ipRGCs, preferentially survive in chronic neurodegenerative conditions. In contrast, ooDSGCs and F-RGCs are among the susceptible RGC subclasses (Figures 1C and 1D). We also adapted an *in vivo* imaging approach to determine the resiliency of αRGCs among RGCs in the SOHU model.^24,25^ We imaged YFP-labeled RGC subsets using scanning laser ophthalmoscopy, which tracked αRGCs *in vivo*, in comparison with a pan-RGC labeling line (Thy1-YFP-17) (Figure S1F). We observed a significant reduction in pan-RGC labeling due to RGC loss, while the αRGCs demonstrated relatively robust and preferentially survival (Figure S1F). Notably, OFF-transient αRGCs are not the RGC subtype that are resilient to SOHU, consistent with past reports.^15^ Our past studies also demonstrated that αRGCs and ipRGCs were among the RGC subclasses resilient to ONC treatment.^7^ Thus, our data suggested that the resilient RGC subclasses under glaucomatous conditions are similar to those under an axotomy setting.

### Resilient αRGCs and ipRGCs demonstrate elevated Spp1 expression after SOHU treatment

**Figure 2.**
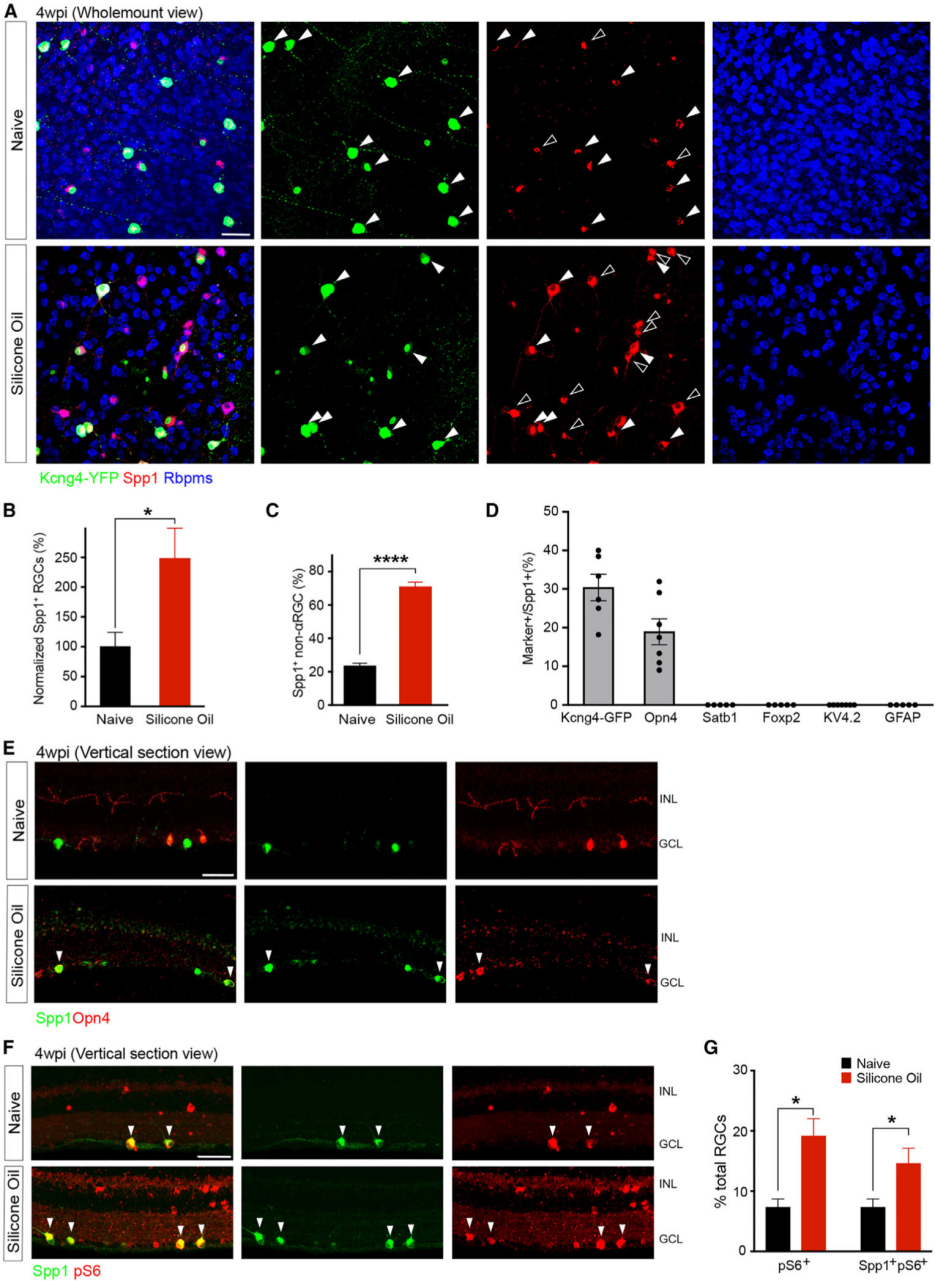
Resilient αRGCs and ipRGCs demonstrate elevated Spp1 expression after SOHU treatment (A) Representative retinal whole-mount images of Kcng4-YFP naive (top) and 4 wpi (bottom), labeled with antibodies to YFP (green), Spp1 (red), and Rbpms (blue). Arrows indicate the overlap of Spp1 and YFP; empty arrowheads indicate ectopic Spp1 expression, which is YFP negative. However, all Spp1 expression is restricted in Rbpms cells, indicating a restricted expression of Spp1 in RGCs. (B) Quantifications of Spp1-positive RGCs numbers in both conditions indicating a significant increase of ectopic Spp1 expression, with the results being normalized to the naive group. n = 5 animals per condition. (C) Quantification of the proportion of non-α-type RGC numbers exhibiting ectopic expression of Spp1 to the number of RGCs positive for Spp1. n = 5 animals per condition. (D) Quantifications of the overlap between Spp1 and other markers for RGC subclasses (representative images shown in Figures S2A–S2E). n = 5–7 animals per condition. (E) Vertical section of the naive retina (top) and 4 wpi (bottom), labeled with antibodies to Spp1 and Opn4 (melanopsin). Arrows indicate the overlap of Spp1 andOpn4 under SO treatment. Green, Spp1; red, Opn4. (F) Vertical section of Kcng4-YFP (αRGCs) naive retina (top) and 4 wpi (bottom), labeled with antibodies to pS6 and Spp1. Arrows indicate the overlap of Spp1 and pS6. Green, Spp1; red, pS6. (G) Fractions of the number of RGCs that have high-pS6-positive levels in both conditions, while the majority of the pS6-positive increase is coupled with Spp1-positive elevation. Scale bars (A, E, and F), 50 μm. n = 5 animals per condition. Unpaired two-sided Student’s t tests; ****p < 0.0001; *p < 0.05.

To investigate Spp1’s potential role in αRGCs resiliency in neurodegeneration settings, we first examined Spp1 expression in the SOHU model. We quantified Spp1 expression levels at 4 wpi and found that Spp1 expression increases with longer exposure to elevated IOP (Figures 2A, 2B, and S2F). In addition, the enhanced Spp1 expression is restricted to Rbpms-positive neurons at the ganglion cell layer (GCL) but not in the inner nuclear layer (INL) (Figures 2E and 2F), suggesting that Spp1 is expressed in response to the SOHU model in RGCs but not in other retina neuron types. In addition, we did not observe detectable Spp1 expression in glial cells in naive or SOHU conditions (Figures 2D, S2D, and S2E). Notably, the elevated IOP led to a chronic increase in Spp1 (Figure S2F), which is different from the rapid downregulation of Spp1 at 3 and 7 days post-crush (dpc) (Figure S2G).

Since the majority of the ectopic Spp1 expression was detected in non-αRGC subsets (Figures 2A–2C), we examined which RGC subclasses possess ectopic Spp1 expression. Spp1 ectopic expression occurred in a large fraction of Opn4-staining-positive RGCs (M1/M2 RGCs; Figures 2D and 2E) but not in several other RGC subclasses, such as ooDSGCs or F-RGCs (Figures 2D and S2A–S2C). We next examined whether the elevated and ectopic Spp1 expression was linked to the activation of the mTOR pathway for RGC neuroprotection.^26^ We measured Phospho-S6 (pS6) levels, an indicator of mTOR activation among the Spp1-positive RGCs. We found that an increase in the pS6-positive RGC number was coupled with an increase in the number of Spp1-positive RGCs at 4 wpi (Figures 2F and 2G). This is in contrast to ONC conditions, where the levels of both pS6 and Spp1 drastically decreased (Figures S2H and S2I). Together, these data suggest that ectopic Spp1 activates mTOR signaling to maintain neuronal resiliency of these RGC subclasses, including both αRGCs and ipRGC subsets. However, Spp1 may play different roles in chronic and acute injuries.

### Spp1 is essential for driving αRGC resiliency in the glaucoma model

**Figure 3.**
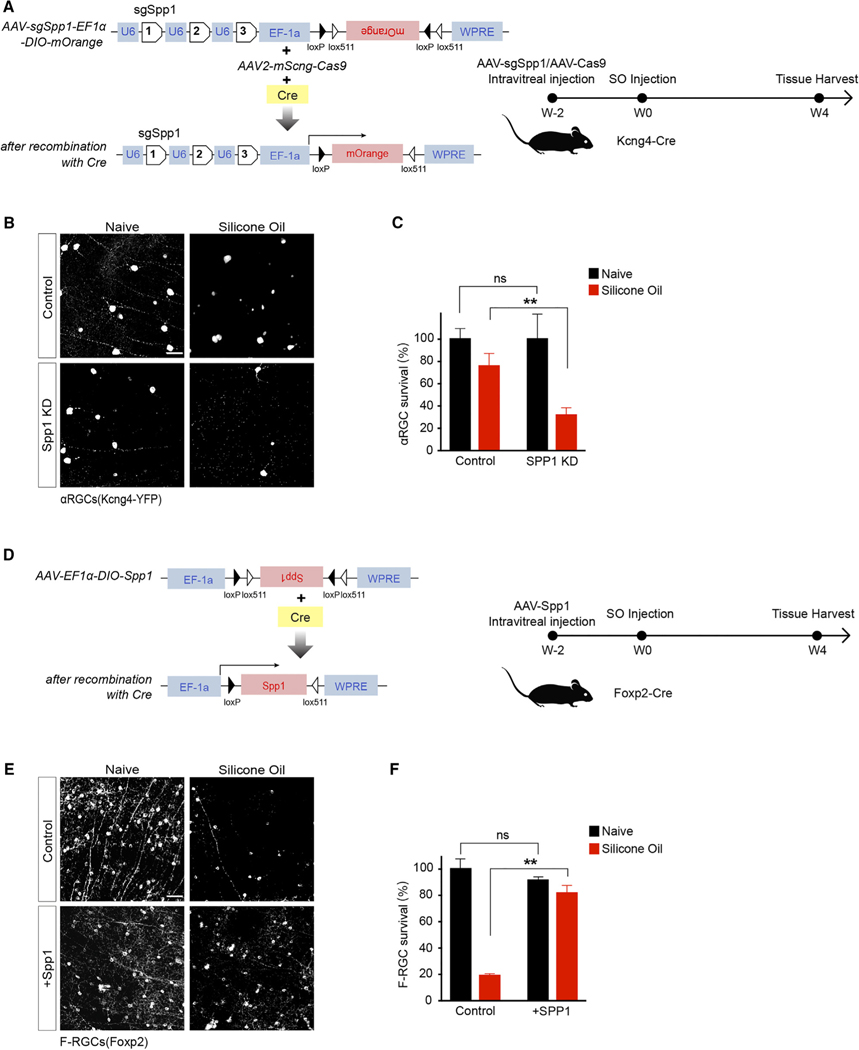
Spp1 is essential for driving αRGC resiliency (A) Schematic of the AAV construct for CRISPR-Cas9-mediated Spp1 knockdown in Kcng4-Cre-positive neurons (left) and timeline of experiment design for knockdown Spp1 at 2 weeks before SO injection and tissue harvest at 4 wpi (right). (B) Control sgRNA/Cas9 Kcng4-YFP retina (top left), retina under SO treatment (top right), sgSPP1/Cas9-infected retina (bottom left), and sgSPP1/Cas9-infectedretina under SO treatment (bottom right), labeled with antibody to YFP. (C) Quantification of normalized αRGC survival. n = 5 animals per condition. (D) Schematic of the AAV construct for Spp1 overexpression in Foxp2-Cre-positive neurons (left) and timeline of experiment design for overexpressing Spp1 at 2 weeks before SO injection and tissue harvest at 4 wpi (right). (E) Control AAV-expression retina (top left), retina under SOHU treatment (top right), AAV-Spp1-infected retina (bottom left), and AAV-Spp1-infected retina under SOHU treatment (bottom right), labeled with antibody to Foxp2. (F) Quantification of normalized F-RGC survival. Scale bars (B and E), 50 μm; n = 5 animals per condition. Paired t test; ns, not significant; **p < 0.01.

Next, we investigated the role of Spp1 in glaucomatous conditions. First, we crossed the αRGC marking line (Kcng4-Cre; LSL-YFP) to an Spp1-null mutant. We found that αRGCs’ survival decreased significantly at 4 wpi (Figures S3G and S3H) from 73% ± 7% to 44% ± 4%. Next, to determine whether the protection was a cell-autonomous effect of Spp1 or whether it was due to the Spp1 expression in other non-αRGCs (e.g., glia),^27^ we generated a selective SPP1 knockout strategy in αRGCs. We injected a combination of AAV-Cas9 and AAV2-expressing gRNAs targeting Spp1 together on Kcng4-Cre; Thy1-stop-YFP mice eye. This led to a marked reduction of αRGC Spp1 levels (Figures S3A and S3B) without any observable change in non-αRGC subclasses. In the SOHU model, this selective loss of Spp1 led to a marked decrease in the viability of αRGCs, dropping from 76% ± 10% to 32% ± 6% (Figures 3A–3C), suggesting that the resiliency of αRGCs depends in part on the cell-autonomous expression of Spp1. Reversely, we asked whether overexpression of Spp1 in otherwise susceptible non-αRGCs results in increased survival. We manipulated F-RGCs given the pronounced loss of F-RGCs in response to chronically elevated IOP (Figure 1C). Foxp2-Cre, in combination with AAV-expressing Cre-dependent-Spp1 (Figures 3D, S3C, and S3D), led to significantly increased F-RGC survivability at 4 wpi (Figures 3E and 3F). In adults, overexpression of Spp1 in F-RGCs does not increase these neurons’ somata sizes (Figures S3E and S3F). These F-RGCs are post-mitotic cells restricted by Foxp2-Cre-dependent overexpression. Thus, the fate changes are likely not happening. Overexpression of Spp1 in these neurons led to significant pS6 elevation. Lastly, we also tested the role of Spp1 in neuroprotection subject to ONC at 14 dpc. In contrast to the Spp1-mediated neuroprotection in the SOHU-treated retina (Figure 3), AAV-mediated Spp1 overexpression under ONC conditions did not lead to significant neuroprotection (Figure S3K). These data suggest that Spp1 plays a key role in driving RGC resiliency in the glaucoma model.

### Spp1 is enriched in adult human RGCs with relatively large somata

**Figure 4.**
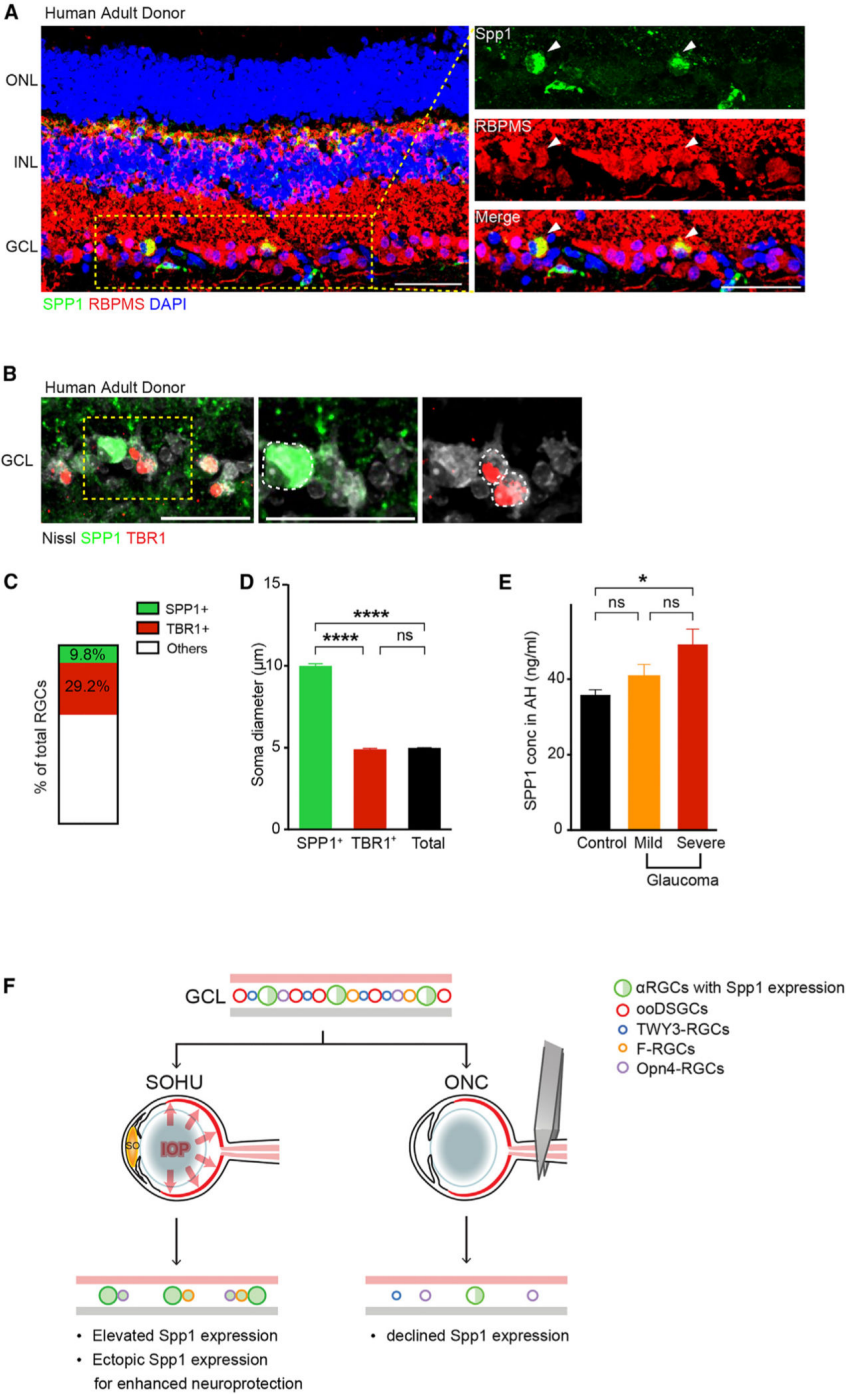
Spp1 is enriched in adult human RGCs with large somata, and SPP1 expression correlates with the glaucoma severity in human patients (A) Sample images of SPP1 (green) and RBPMS (red) in the retina from an adult donor sample eye with no notable ocular history, including expression in RGC and horizontal cell subsets. (B) Sample images of SPP1 (green), TBR1 (red, a previously characterized transcription factor found in T-RGCs/midget OFF-RGCs),^28^ and Nissl (gray) in the retina from a donor in (A). (C) Percentage of SPP1-positive and TBR1-positive RGCs in the adult human retina from the donor in (A) and (B). n = 2 retinas. (D) Quantification of RGC soma size of SPP1-positive, TBR1-positive, and the average Nissl (NeuroTrace)-positive RGCs in the adult human retina. n = 2 retinas. (E) SPP1 concentration in aqueous humor was quantified using ELISA from patients with mild (n = 11) and severe (n = 13) forms of primary open-angle glaucoma (POAG) and age-matched controls (n = 16). Significance was assessed by one-way ANOVA followed byTukey’s Honest Significant Difference (HSD) test for pairwise comparisons, demonstrating a significant difference between patients with severe glaucoma and control patients (p = 0.02). AH, aqueous humor. (F) Model illustrating the differential actions of Spp1 and survival of different RGC subclasses between SOHU and ONC treatments. In the SOHU model, Spp1 expression is upregulated, and ectopic Spp1 expression provides protection for RGCs. In the ONC model, Spp1 expression is downregulated, and restricted Spp1 expression in αRGCs only provides limited protection for αRGCs. Scale bars (A and B), 50 μm.

To investigate whether similar mechanisms are found in the human retina, we stained SPP1. We detected adult RGC subsets expressing SPP1 (8.7% ± 2%) in freshly preserved human donor retina samples (Figures 4A–4C), including the macular and the periphery (Figures S4B and S4C). SPP1 also labeled a horizontal cell subset (Figure S4G).^28^ We used RBMPS to mark all RGCs and TBR1 to mark a previously characterized human RGC subset.^28^ We found that the SPP1-positive human RGC somata sizes are significantly larger than other RGCs, including TBR1-positive RGCs (Figures 4B–4D). We analyzed SPP1 expression in the human prenatal retina at gestational week (GW) 22–23 (i.e., the end of retinal neurogenesis)^29^ and did not detect SPP1 expression at the GCL; however, TBR1 expression in RGCs was abundant at this prenatal stage (Figure S4D), in contrast to the SPP1 and TBR1 in adults (Figures 4B, S4B, and S4C). SPP1 is enriched in mature human RGC subsets. Notably, SPP1-positive RGCs belong to a subset of SMI32-positive human RGCs (~18%), an established marker for the human RGC subset, with relatively large somata (Figure S4A).^30^ SPP1 expression is not detected in GFAP-positive human astrocytes (Figure S4E).

### SPP1 expression correlates with the severity of glaucomatous neuropathy in human patients

Elevated Spp1 expression in the mouse glaucomatous retina led us to explore whether this is also true in the human retina, especially during disease etiology. Due to limited access to the retinal tissue of patients with glaucoma, we took advantage of the fact that SPP1 is a secreted protein that can be detected in aqueous humor (AH). We acquired AH samples from individuals undergoing cataract surgery—patients without a history of glaucoma served as controls, while individuals with mild or severe primary open-angle glaucoma (POAG) provided the glaucomatous samples. We measured the SPP1 level in AH using ELISA. The patients in these three groups were similar in age, gender, race, and lens status (Table S1). Those with severe disease had a higher mean IOP than the mild POAG and control groups. Patients with mild POAG and controls had a similar SPP1 level, while patients with severe POAG had significantly elevated SPP1 concentration (Figure 4E). These human data indicate a consistent correlation between SPP1 and glaucomatous optic neuropathy. Furthermore, these data illustrated that SPP1 may serve as an adult biomarker for disease progression in patients with glaucoma.

## DISCUSSION

Our study revealed Spp1 as a critical molecular player driving αRGC-specific neuroprotection in glaucomatous optic neuropathy. Spp1 protein levels increase during prolonged IOP elevation in mice. Furthermore, loss-of-function and gain-of-function studies in genetically distinct RGC subsets suggested that Spp1 drives neuronal resiliency in SOHU, but not ONC, conditions. These data point to a model whereby increased Spp1 expression in αRGCs under chronic glaucomatous insult results in αRGC resiliency (Figure 4F). Analysis of AH from patients with glaucoma showed that SPP1 expression is also relevant to humans with glaucomatous neuropathy.

Mechanistically, Spp1 is characterized as a member of the matricellular protein family, an important regulator of extracellular matrix mineralization, and has proven to be a strong marker of calcification and vascular diseases.^31–34^ The specific mechanism of the Spp1 signaling cascade in retinal neurons, particularly in αRGCs and ipRGCs known for their neuronal resiliency, has yet to be thoroughly investigated. Notably, a recent study explored a non-neuronal expression of Spp1 in astrocytes under various optic neuropathies and natural aging conditions.^27^ However, while we did not detect Spp1 expression derived from glial cells in either naive or SOHU conditions, we did observe a highly regulated Spp1 expression specific to RGC neurons (Figures S2D and S2E). The observation stays the same in humans, where SPP1 expression is dominant in human retinal neurons, including RGCs, but not in astrocytes (Figure S4E). The neuroprotection strategy utilized in this study (Figure 3) and in Li and Jakobs^27^ was known to target RGCs but not astrocytes via AAV serotype 2. Spp1 receptors include CD44 and several specific integrins.^35,36^ CD44 is expressed in retinal muller glia but not in neurons (Figure S2J). The other well-characterized receptor for Spp1 was ItgaV (not identical to Itga5, an integrin member characterized by Li and Jakobs^27^ but not known as an Spp1 receptor).^37,38^ ItgaV is expressed in RGCs, including αRGCs, but not in astrocytes (Figures S2K–S2M). The expression patterns of CD44 and ItgaV offered potential targets to explore neuronal Spp1-mediated neuroprotection.

Our study offers several insights: first, our studies’ differences between ONC and SOHU models suggested Spp1’s role in promoting RGC resiliency in mouse models of glaucomatous neuropathy but not axotomy (Figure 4F). This difference may reflect the eyes’ attempt to promote neuroprotection, with subtypes of RGCs possessing elevated Spp1 expression and increased resilience potentially driving this impact amid chronic neurodegeneration. Second, immunostaining identified a subset of SPP1expressing, SMI32-positive RGCs with relatively large somata in the adult but not the prenatal human retina. This correlates with mouse Spp1 expression in adult αRGCs but not in developing retinas.^7^ The timing of expression during development also indicates a potential role for SPP1 in neuronal maturation and somatic size regulation. The αRGC subclass, particularly the αRGC transient subtype, is physiologically similar to Y-RGCs in primates due to their non-linear receptive fields,^39,40^ suggesting evolutionarily conserved roles for the RGC subclass. Enabled by the utilization of promptly collected human pathology samples to maintain both RGC function and transcriptomes, future work may correlate these SPP1-expressing human RGC subsets to human RGC subtypes defined by single-cell RNA sequencing (RNA-seq),^28,41^ as well as subtypes defined by physiology, such as Y-RGCs with non-linear receptive fields with either ON or OFF light responses. Last, in the human ocular disease setting, the correlation of SPP1 levels with the severity of optic neuropathy in patients with glaucoma raises the possibility that SPP1 may be a relevant biomarker and a potential therapeutic target for glaucoma. Several past studies assessed SPP1 levels in POAG and primary angle-closure glaucoma with differential findings.^42,43^ Further characterization of SPP1 levels in humans with progressive optic nerve damage may help in the decision to escalate medical or surgical treatment. Assessing SPP1 levels in a larger cohort of patients with varying glaucoma severities, varying treatment interventions, and over an extended treatment course would help refine the diagnostic utility of such a biomarker.

### Limitations of the study

There were several limitations of our current study. First, we proposed that secreted Spp1 acts in an autocrine or paracrine manner onto multiple receptors, including ItgaV (Figures S2K–S2M), which mediate the neuroprotective actions. However, the intracellular Spp1’s roles were not ruled out.^44^ Second, the mechanisms underlying ipRGC-specific neuroprotection remain to be explored, especially considering their distinct responses to both ONC and SOHU treatments and the roles of ipRGC-derived molecules in mediating neuroprotection.^10^ Third, the current SOHU model is not adaptable to regeneration studies due to the nature of its chronic insults and the indistinguishable regenerating axons and spared axons. Thus, it is hard to define the zero time point for the regeneration and evaluate optic nerve regrowth abilities under a glaucomatous setting. Last, the source of SPP1 in human AH may not solely come from neuronal secretion, especially considering the non-neuronal cells expressing SPP1 in the anterior segments.^45^

## STAR★METHODS

### RESOURCE AVAILABILITY

#### Lead contact

Further information and requests for resources and reagents should be directed to and will be fulfilled by the Lead Contact, Xin Duan (xin.duan@ucsf.edu).

#### Materials availability

Materials used in this study will be provided upon request and available upon publication.

#### Data and code availability

- All data reported in this paper will be shared by the lead contact upon request.
- This paper does not report the original code.
- Any additional information required to reanalyze the data reported in this paper is available from the lead contact upon request.

### EXPERIMENTAL MODEL AND SUBJECT DETAILS

#### Mice

All animal experiments were approved by the Institutional Animal Care and Use Committees (IACUC) at the University of California at San Francisco, Stanford University, and the University of California at San Diego. Mice were maintained under regular housing conditions with standard access to food and drink in a pathogen-free facility. Male and female mice were used in roughly equal numbers; no sexual dimorphisms were observed. Animals with noticeable health problems or abnormalities were not used. All ages and numbers were documented. Genotypes were determined by PCR from the tail biopsy. The following mouse lines were used: Kcng4-Cre, Foxp2-Cre, Opn4-GFP, Cartpt-Cre, TWY3-YFP, Thy1-STOP-YFP, Thy1-YFP17 and Spp1-KO.

1. Kcng4-Cre (Jax: 029414) mouse line. The Kcng4^Cre^ knock-in allele was designed to both abolish endogenous gene function and allow the Kcng4 promoter/enhancer regions to direct Cre recombinase expression to subsets of alpha retinal ganglion cells (α-RGCs), as well as Type 5 ON bipolar cells (BC5s).^47^.
2. Foxp2-Cre (Jax: 030541) mouse line. IRES-Cre is expressed from the mouse Foxp2 promoter in a subset of Foxp2-expressing retinal ganglion cells (F-RGCs).^21^.
3. OPN4-Cre (Jax: 035925) mouse line. Opn4^Cre^ knock-in mice express Cre recombinase under the direction of the Opn4 promoter in Melanopsin-expressing retinal ganglion cells (ipRGCs).^48^.
4. Cartpt-IRES-Cre (Jax: 028533) mouse line was a gift from Hongkui Zeng (Allen Institute) and was previously established for studies of retinal ganglion cell subclasses.^19^ Cartpt-Cre marks conventional ON-OFF direction-selective ganglion cells (ooDSGCs) of multiple types, including Hb9-GFP, a conventional Cartpt-positive ooDSGCs.
5. TWY3 (Jax: 033114) mouse line: transgenic mice express YFP under the direction of the neuron-specific mouse Thy1 genepromoter. YFP is detected in a distinct subset of retinal ganglion cells, all of which share a dendritic lamination pattern: dense dendritic arbors are primarily localized to the central third (S3) of the inner plexiform layer.^16^.
6. Thy1-STOP-YFP (Jax: 005630) mouse line. Thy1-STOP-YFP mouse possesses *loxP* sites flanking the STOP codon between the promoter and eYFP gene.^49^.
7. Thy1-YFP17 transgenic mice^50^ label most RGCs (with only a few amacrine cells).
8. Spp1 knockout mice (Jax:004936): The exons 4–7 of the *Spp1* gene were replaced by the *Neo* cassette to generate null allele.^51^.

#### Human retinal tissues

Human adult eye retina tissues were collected strictly following Research Autopsy Collaboration (RACS, IRB 63818, approved by Administrative Panels for the Protection of Human Subjects, Research Compliance Office at Stanford University. Prenatal eye retinal tissues were collected strictly following Protocols (10–05113) and were approved by the Human Gamete, Embryo, and Stem Cell Research Committee at the University of California, San Francisco. Informed consent was obtained from all subjects. De-identified second-trimester human eye tissue samples were collected with previous patient consent in strict observance of the legal and institutional ethical regulations. Human prenatal retinal tissues were harvested from careful eye enucleations, followed by 4% P.F.A. fixation at 4°C overnight. tissues were removed and embedded for long-term storage and subsequent processing.

### METHOD DETAILS

#### Induction of ocular hypertension

Ocular hypertension was induced on all the mouse lines above with one of two methods.

1. Silicone oil-induced ocular hypertension under-detected (SOHU) model^18^: Mice were anesthetized with an intraperitoneal (IP) injection of 2,2,2-tribromoethanol. The intraocular pressure of both mouse eyes was measured after confirming anesthesia. The anesthetized mouse was placed lateral to the operating table, and a drop of procaine hydrochloride 0.5% (Akorn, Somerset, New Jersey) was applied to the cornea before injection to reduce sensitivity. The cornea was punctured with a 32G needle from the superior temporal side (approximately 0.5 mm from the limbus); the needle penetrates about 0.3mm, avoiding damage to the lens or iris. The needle slowly removes some aqueous humor (approximately 1–2μL) from the anterior chamber. A self-made glass microelectrode prefilled with silicone oil (SO, 1,000 mPa s, Silikon, Alcon Laboratories) was inserted through the punctured corneal tunnel. SO was slowly injected into the anterior chamber until the SOHU droplets covered most of the iris surface. The micropipette was held for 30 s, then slowly pulled out. The corneal incision was closed by gently pushing the upper eyelid of the mouse. Antibiotic ointment (B.N.P. antibiotic ophthalmic ointment) was applied to the cornea post-procedure, and during the procedure, artificial tears were used to moisten the cornea. For each SOHU-treated animal included in the dataset, we utilized the following criteria: (1) weekly ocular pressure checks; the IOP was within the range of (24 ± 10 mmHg) over 4 weeks of treatment; (2) 4 weeks of SOHU treatment led to a consistent loss of half of the RGCs as an internal control (Rbpms-positive, Figures 1C and S1B); (3) contralateral retinas collected were devoid of any deficits and variability due to genetics; (4) photoreceptor layers stayed largely intact after glaucomatous conditions to avoid secondary effects of ischemic conditions, potentially caused by hyper-elevated IOP.^52^.
2. Microbead occlusion model: Detailed procedures are described by.^13^ Briefly, mice were placed in the isoflurane chamber to initiate anesthetization. When the mouse was sufficiently anesthetized, the flow of isoflurane was diverted to its nose cone, and the moused was placed on the surgical platform with its nose secured within the cone. The ophthalmic solution, 1% tropicamide (Bausch & Lomb, Tampa, FL), and anesthetic drops (0.5% proparacaine hydrochloride; Bausch & Lomb) were applied topically to the cornea to initiate pupil dilation. Once the pupil was dilated, the eye of the mouse was fully exposed with forceps under the microscope, and a pre-pulled glass micropipette (1.0/0.75 mm OD/ID with filament; World Precision Instruments, Sarasota, FL) filled with microbeads (15-μm polystyrene) was inserted into the anterior chamber from 3 mm central to the ora serrata; 1μL of microbead solution was then injected into the mouse anterior chamber. After injection, the antibiotic ointment was placed on each eye. One eye of each mouse received silicone oil injection or microbeads injection at 8–10 weeks of age. The contralateral eye was injected with an equivalent volume of sterile physiologic saline (Fisher Scientific, Fair Lawn, NJ). As such, each animal serves as its control. The mice were euthanized to obtain tissues 1 week and 4 weeks post-injection of silicone oil or microbeads.

#### Intra-ocular pressure (IOP) measurement

The IOP was monitored once weekly until 4–8 weeks after SOHU or microbeads injection using the TonoLab tonometer (Colonial Medical Supply, Espoo, Finland) according to product instructions. Mice were anesthetized with an I.P. injection of ketamine, xylazine, and acepromazine (70, 10, and 2 mg/kg, respectively). The TonoLab tonometer measures five times, removes high and low readings, and produces an average result. We considered this machine-generated average as one reading. The IOP of each eye was determined by averaging three machine-generated readings taken every 5 min. The cornea was moistened with artificial tears during this procedure.

#### Intravitreal injection

Mice were anesthetized with ketamine/xylazine/acepromazine (70/10/2 mg/kg). For intravitreal injection, AAV (0.5μL–1μL) was injected into the posterior chamber through the point directly behind the limbus (beneath the iris) with a fine glass pipette 2 weeks before induction of ocular hypertension.

#### Histology

A lethal overdose of anesthesia sacrificed the mice. The eyes were dissected and post-fixed with 4% PFA. on ice for 1 h and rinsed with PBS. Retinas were analyzed as cryosections and whole mounts. For frozen sections, tissues were immersed in 30% sucrose for 2 h, then frozen in OCT before sectioning in a cryostat (20μm). For immunohistochemistry, sections were incubated in PBS with 3% donkey serum and 0.3% Triton X-100 for 1-h blocking, followed by primary antibodies overnight at 4°C. For wholemount retinas, tissues were incubated with blocking buffer (5% normal donkey serum, 0.5% Triton X-100 in PBS) overnight, followed by primary antibodies for 2–4 days at 4°C. Secondary antibodies were applied for 2 h at room temperature. Finally, sections and wholemount retinas were washed with PBS and mounted onto glass slides using SlowFade Gold antifade reagent (Invitrogen).

Primary antibodies used were as follows (and detailed in STAR Methods Table): chicken anti-GFP (1:1000, Abcam); guinea pig anti-RBPMS (1; 1000, Phospho Solutions); rabbitanti-RBPMS(1:1000, Proteintech); goatanti-Osteopontin/Spp1(1:500,R&D Systems); rabbit anti-Melanopsin (1:500, ATSbio); rabbit anti-phosphorylated S6 [Ser235/236](1:200, Cell Signaling Technology); rabbit anti-RFP (1; 1000, Rockland); rabbit anti-Foxp2 (1:1000, Abcam); rabbit anti-Tbr1(1:500, Abcam); rabbit anti-Satb1 (1:1000, Abcam); mouse anti-Kv4.2 (1:200, UC Davis/NIH NeuroMab Facility); mouse anti-neurofilament (SMI32, 1:1,000, Convance); rat anti-Integrin alpha V(1:500, Abcam); mouse anti-GFAP(1:250, Sigma-Aldrich); rat anti-CD44 (1:200, Millipore); mouse anti-Glutamine synthetase (1:1000, BD Transduction Labratories); rabbit anti-Calbindin (1:100, Swant). Nuclei were labeled with NeuroTrace (Nissl 435/455, 1:1000, Invitrogen). The primary antibodies were detected with Alexa Fluor 488, Alexa Fluor 568, and Alexa Fluor 633 (1:1000, Invitrogen).

#### Adeno-associated viral (AAV) vectors

AAV serotype 2/2 was produced using a previously validated method for *in vivo* application, which dominantly infects neurons at GCL toward RGCs.^7^ We transfected re-engineered vectors, together with pXX860 (helper plasmid) and pAAV2 (viral capsid), into 10 plates of 150mm dishes of 293FT cells (Invitrogen). We harvested the triple-transfected cells 3 days post-transfection and purified AAV2 using the Iodixanol gradient ultracentrifugation method. AAV2 will be titered to >1×10^12^ G.C./mL based on a qPCR method before *in vivo* applications.

1. Intraocular injections of the following AAV2 constructs were adopted for neuronal-type labeling or manipulations as previouslyestablished.^7^ AAV-EF1a-BbChT (a gift from Dawen Cai & Joshua Sanes, Addgene # 45186) was intravitreally injected into Foxp2-Cre, Cartpt-Cre, or Opn4-cre mice with 1.5μL AAV (in 1x DPBS, AAV was standardized to the same titer of 2×10^12^ G.C./mL) to label F-RGCs, ooDSGCs, and ipRGCs, respectively. AAV-EF1a-DIO-Spp1 (Figure 3D) was intravitreally injected into Foxp2-Cre mice to overexpress Spp1 on F-RGC.
2. AAV-CRISPR/Cas9-mediated depletion of Spp1: The expression cassette of multiple sgRNA targeting Spp1 was constructed by the multiplex CRISPR/Cas9 assembly system (Addgene#1000000052). We designed the 3 sgRNA sequences for Spp1 (NCBI) using CHOPCHOP (https://chopchop.cbu.uib.no). Then these short oligos were assembled to the pX330A_D10A-1×3 plasmid, followed by the kit’s protocol. To make AAV, we amplified the cassette, including three pairs of U6 promoter and sgRNA by PCR, and ligated it to AAV-Ef1a-DIO-mOrange2 plasmid (Figure 3A).

The sequences of sgRNAs are below: sgRNA1: 5’- CCTACAGTCGATGTCCCCAA-3′, sgRNA2: 5’- ATCGATCACATCCGACTGAT-3′, sgRNA3: 5’- CGTTGGGGACATCGACTGTA-3’. The sgRNA-non-targeting aka sgRNA1: 5′-AACGACTAGTTAGGCGTGTA-3′, sgRNA2: 5′-GAACGACTAGTTAGGCGTGTA-3′, sgRNA3: 5′-GTTGGAGCACTGTCCTCCGAACGT-3’ (targets Gal4 sequence) as published^53,54^ were adopted in AAV-sgRNA-DIO- mOrange2 plasmid. 1μL of AAV-sgSpp1-Ef1a-DIO-mOrange2 or AAV-sgRNA-non-targeting was intravitreously injected with 1μL of AAV-mSncg-Cas9.^46^ AAV-sgSpp1-Ef1a-DIO-mOrange2 was standardized to 1.5×10^12^ G.C./mL in 1x DPBS, while AAV-mSncg-Cas9 was standardized to 3×10^12^ G.C./mL in 1x DPBS.

#### Human aqueous humor fluid for SPP1 Measurement

Human aqueous fluids were collected strictly following Protocols (17–22840), approved by the University of California San Francisco Institution Review Board, and adhered to the Declaration of Helsinki for research involving human subjects. Written informed consent was obtained from all patients. Clinical data were extracted from the records and collated in a fully anonymized manner. Aqueous fluid was collected during the surgeries. A paracentesis into the anterior chamber was performed before injecting any intraocular agent, and 50–100 μL aqueous fluid was collected through the paracentesis using a 27-gauge needle. Caution was taken to ensure no blood or cellular debris contamination. All aqueous fluid samples were then labeled with a predetermined identification number. Samples were then transferred to the research laboratories on dry ice and stored at −80°C until processing.

SPP1 was quantified in patients undergoing cataract surgery without a history of glaucoma or any other intraocular pathology (Controls; n = 16) or patients undergoing glaucoma surgery (Ahmed valve implantation, micro-invasive glaucoma surgery, trabeculectomy combined with or without cataract extraction and intraocular lens placement) from December 2017 to September 2020. Glaucoma patients were divided into those patients with mild (n = 11) or severe (n = 13) primary open-angle glaucoma (POAG). Mild POAG was defined based on the Humphrey Visual Field mean deviation (MD) between −1 and −5, while the severe POAG group included those patients with an MD less than −10. SPP1 levels were quantified using the human SPP1 enzyme-linked immunosorbent assay (ELISA) kit (DOST00, R&D Systems, Minnesota) using a 50-fold dilution of aqueous fluid from patients and following the manufacturer’s instructions. Fluorescence at 450 nm was determined using a microplate reader, and the final Spp1 concentration (ng/mL) was calculated based on a standard curve of recombinant SPP1 (See below for statistics details).

### QUANTIFICATIONS AND STATISTICAL ANALYSIS

#### Data acquisition

Confocal images were acquired using a Leica SP8 confocal microscope (Leica Microsystems) and Zeiss LSM900 (Carl Zeiss Microscopy). For both retinal wholemounts and sections, images were taken with a step size of 1μm (wholemounts) or 0.5μm (sections) and a 20X, 40X, or 63X lens. For wholemounts of retinas, at least eight areas (~0.5 × 0.5 mm) were imaged across the whole retina, including two on each nasal, temporal, ventral, and dorsal side. One field from at least eight sections per sample was imaged and analyzed for retinal sections. The numbers from all sections were averaged to generate a single value for each retina. Images were analyzed using ImageJ software (NIH). Only contrast and brightness were adjusted for all images. Images were carefully not oversaturated, and only cells that stained brightly were counted when positive staining was to be determined.

#### Statistical analyses

ImageJ was used to process all images. GraphPad Prism 9 was used to generate graphs and statistical analyses. Statistical methods and the number of animals tested with each configuration are presented in the legend for each figure; for comparisons between two groups, paired or unpaired two-sided Student’s t-tests or Fisher’s exact tests were used as indicated in the figure legends. A one-way analysis of variance (ANOVA) was used for the statistical analysis of multiple groups. Statistical significance was defined as ns, not significant, p < 0.05(*), p < 0.01(**), p < 0.001(***), and p < 0.0001 (****). All Data are presented as means ± s.e.m unless stated otherwise.

1. To quantify the survival of RGCs: The retina images were acquired from at least eight sections as described above using 20Xmagnification. The number of GFP-positive neurons or marker-positive cells from all stained samples was counted. A single value for each retina was obtained by averaging the counts obtained from all areas. Confocal stacks of the whole RGC layer were acquired and processed to obtain maximum intensity projections. Each subclass baseline was represented as 100%. Comparisons between the baseline and two post-injection time points (1wpi and 4wpi) within each subclass were analyzed using unpaired two-sided Student’s t-tests. Comparisons between SOHU, microbeads injected or crushed eyes, and contralateral eyes were analyzed using unpaired two-sided Student’s t-tests.
2. To quantify the differences in IOP at each timepoint: IOP of both eyes was monitored using TonoLab pre-injection and weekly until 4–8 weeks post-injection. The IOP of each mouse will be measured 6 times at each time point, and the average value will be taken. The number per condition at each time point was stated as in legends. The graphs and statistical analysis were generated by GraphPad Prism 9.
3. To quantify the Spp1 expression after SO/microbeads treatment, Spp1 overexpression and knocking down: Confocal images of retina wholemounts were taken by Leica SP8 at 20× magnification lens with z-step of RGC layer after SPP1 staining. The number of SPP1-positive cells was counted. We normalized the counts using the control group as the baseline. Comparisons between control and treatment groups were analyzed using unpaired two-sided Student’s t-tests.
4. To quantify the Spp1-positive non-αRGC cell numbers: Leica SP8 acquired source retina wholemounts images with 20× magnification lens and z stack. Kcng4-YFP positive cells were treated as αRGC-positive cells. The cells that were Spp1-positive, but Kcng4-YFP-negative were counted. In addition, immunohistochemistry markers (such as Melanopsin, Kv4.2, Foxp2, and Tbr1) were also quantified in Figure S2. Comparisons between control and SOHU groups were analyzed using unpaired two-sided Student’s t-tests.
5. To count Spp1 and pS6 positive cells: Retina sections images were acquired by Leica SP8 with 203 magnification lens and z stack. Spp1 and pS6 positive cells were counted from at least eight sections per sample. Comparisons between control and SOHU groups were analyzed using unpaired two-sided Student’s t-tests.
6. To quantify the percentages of SPP1-positive and TBR1-positive and soma size in the human retina: NeuroTrace Nissl stain was used to label all RGCs. Zeiss LSM900 took the section images of the central retina with a 20× magnification lens. Spp1 and Tbr1 positive cells were counted from eight sections per retina. The number of total RGCs was generated from NeuroTrace Nissl-positive cells. Z stacks were projected onto a single plane, and the largest area was measured for soma size with ImageJ.
7. Quantification of SPP1 in human aqueous fluid: Spp1 was quantified in patients undergoing cataract surgery without a history of glaucoma or any other intraocular pathology (controls; n = 16) or patients undergoing glaucoma surgery (Ahmed valve implantation, micro-invasive glaucoma surgery, trabeculectomy combined with or without cataract extraction and intraocular lens placement) from December 2017 to September 2020. Patients with glaucoma were divided into those patients with mild (n = 11) or severe (n = 13) primary open-angle glaucoma (POAG). Mild POAG was defined based on the Humphrey Visual Field mean deviation (MD) between −1 and −5, while the severe POAG group included those patients with an MD less than −10. The three groups were indistinguishable based on age, sex, race, laterality, and lens status (Table S1). Statistical significance was determined using a one-way analysis of variance (ANOVA) followed by Tukey H.D. for pairwise comparisons and in R 4.0.1 (R Core Team 2020).

## Supplementary Material

1

## Figures and Tables

**Table:** KEY RESOURCES TABLE

REAGENT or RESOURCE	SOURCE	IDENTIFIER
Antibodies		

chicken anti-GFP	Abcam	Cat#ab13970; RRID:AB_300798
guinea pig anti-RBPMS	Phospho Solutions	Cat#1832-RBPMS; RRID:AB_2492226
rabbit anti-RBPMS	Proteintech	Cat#15187–1-AP; RRID:AB_2238431
goat anti-Osteopontin/Spp1	R&D Systems	Cat#AF808; RRID:AB_2194992
rabbit anti-Melanopsin	ATSbio	Cat#AB-N38; RRID:AB_1608077
rabbit anti-phosphorylated S6 [Ser235/236]	Cell Signaling Technology	Cat#4856; RRID:AB_2181037
rabbit anti-Foxp2	Abcam	Cat#ab16046; RRID:AB_2107107
rabbit anti-Tbr1	Cell Signaling Technology	Cat#49661; RRID:AB_2799364
rabbit anti-Satb1	Abcam	Cat#ab109122; RRID:AB_10862207
mouse anti-Kv4.2	UC Davis NeuroMab Facility	Cat#75–361; RRID:AB_2315874
mouse anti-neurofilament (SMI32)	Biolegend	Cat#801701; RRID:AB_2564642
mouse anti-GFAP	Sigma-Aldrich	Cat#G3893; RRID:AB_477010
rabbit anti-Calbindin	Swant	Cat#CB38; RRID:AB_10000340
rat anti-Integrin alpha V	Abcam	Cat#ab63490; RRID:AB_1140041
rabbit anti-RFP	Rockland	Cat#600–401-379; RRID:AB_2209751
NeuroTrace^™^ 435/455 Blue Fluorescent Nissl Stain	Invitrogen	Cat#N21479
rat anti-CD44	Millipore	Cat#217594; RRID:AB_2076209
mouse anti-Glutamine Synthetase	BD Transduction Laboratories	Cat#610518; RRID:AB_397880

Bacterial and Virus Strains		

AAV-EF1a-BbChT	Addgene	Cat#45186
AAV-EF1a-DIO-Spp1	Boston Children’s Hospital Viral Core	N/A
AAV-mSncg-Cas9	Stanford Ophthalmology Viral Core	Wang et al.^46^
AAV-sgSpp1-Ef1a-DIO-mOrange2	This study	N/A
AAV-sgRNA-non-targeting Ef1a-DIO-mOrange2	This study	N/A

Biological Samples		

Mouse eye tissues	Mouse strain is listed in the “Experimental Models: Organisms/Strains.”	N/A
Human adult eye retinal tissues	Research Autopsy Collaboration (RACS) at Stanford University	IRB#63818
Human prenatal eye retinal tissues	Human Gamete, Embryo, and Stem Cell Research Committee at the University of California, San Francisco	IRB#10–05113
Human aqueous humor fluid	University of California San Francisco, Department of Ophthalmology	IRB#17–22840

Chemicals, Peptides, and Recombinant Proteins		

Silicone oil	Alcon Laboratories	1,000 mPa s, Silikon

Experimental Models: Organisms/Strains		

Mouse: C57BL/6J (WT)	Jackson Laboratory	Cat# 000664
Mouse: Kcng4-Cre	Jackson Laboratory	Cat# 029414
Mouse: Foxp2-Cre	Jackson Laboratory	Cat# 030541
Mouse: Opn4-Cre	Jackson Laboratory	Cat# 035925
Mouse: Cartpt-IRES-Cre	Jackson Laboratory	Cat# 028533
Mouse: TWY3	Jackson Laboratory	Cat# 033114
Mouse: Thy1-STOP-YFP	Jackson Laboratory	Cat# 005630
Mouse: Spp1 KO	Jackson Laboratory	Cat# 004936

Software and Algorithms		

Graphpad Prism9	https://www.graphpad.com/	RRID: SCR_002798
Fiji	https://fiji.sc/	RRID: SCR_002285

## Highlights

- αRGCs and ipRGCs are resilient retinal neuron types in glaucomatous and axotomy conditions
- Elevated and ectopic Spp1 expression among RGCs occurs in the glaucoma model, not axotomy
- Neuronal Spp1 drives RGC neuroprotection in glaucomatous conditions but not in axotomy
- SPP1 protein levels in aqueous humor correlate with glaucoma progression in patients

## INCLUSION AND DIVERSITY

One or more of the authors of this paper received support from a program designed to increase minority representation in science.
