# Supplementary material for: Osteopontin drives retinal ganglion cell resiliency in glaucomatous optic neuropathy
Source: Cell Rep. Author manuscript; Available in PMC 2023 Oct 23. (PMC10591811; doi:10.1016/j.celrep.2023.113038)
Supplement: 1 [file NIHMS1933884-supplement-1.pdf]

Cell Reports, Volume *42*

Supplemental information

Osteopontin drives retinal ganglion cell resiliency in glaucomatous optic neuropathy

Mengya Zhao, Kenichi Toma, Benyam Kinde, Liang Li, Amit K. Patel, Kong-Yan Wu, Matthew R. Lum, Chengxi Tan, Jody E. Hooper, Arnold R. Kriegstein, Anna La Torre, Yaping Joyce Liao, Derek S. Welsbie, Yang Hu, Ying Han, and Xin Duan

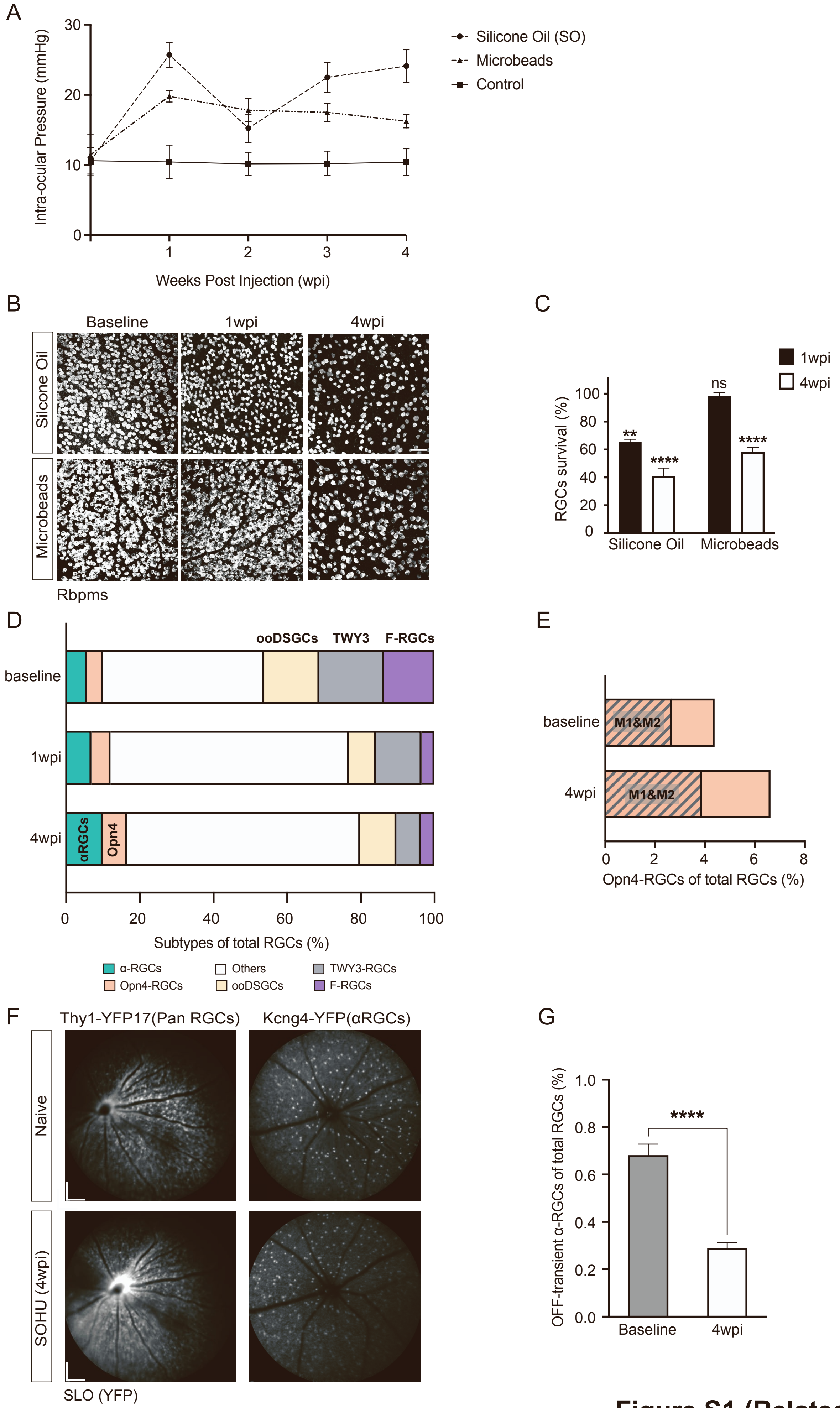

Figure S1 (Related to Figure 1)

Figure S1. An evaluation of the silicone oil and microbead occlusion models with similar RGC survival outcomes (Related to Figure 1).

(A) Intraocular pressure (IOP) levels in the SOHU model, microbeads occlusion model, and naïve retinas. IOP data were collected at consistent time intervals after injection. **(B)** Sample images of wholemount views of retinas labeled with antibodies to Rbpms during silicone oil (top) and microbeads occlusion models (bottom) at different time points. **(C)** Quantification of pan-RGCs survival normalized to the baseline of each condition (100%). n=5 animals per model. Data are presented as means ± s.e.m. The quantifications were generated by comparing 1wpi or 4wpi with the baseline. Scale bar, 50μm. "wpi", weeks post-injection. Unpaired t-test; "ns", not significant; "**", p<0.01; "****", p<0.0001. **(D)** Fraction of all RGCs comprised by each subtype at baseline, 1wpi, and 4wpi. **(E)** Fraction of M1/M2-RGCs (Melanopsin-positive) in the intact retina and 4wpi indicated their preferential survival subject to SOHU. **(F)** Representative SLO retina fundus images of pan RGCs (Thy1-YFP17, left) and αRGCs (Kcng4-YFP, right) from naïve retina (top) and 4 wpi (bottom). Scale bar, 1000μm. (**G**) Percentages of OFF-Transient αRGCs at baseline and 4wpi indicated that they are not among the resilient RGC subtype subject to SOHU. n=5 animals. Unpaired t-test; "****", p<0.0001.

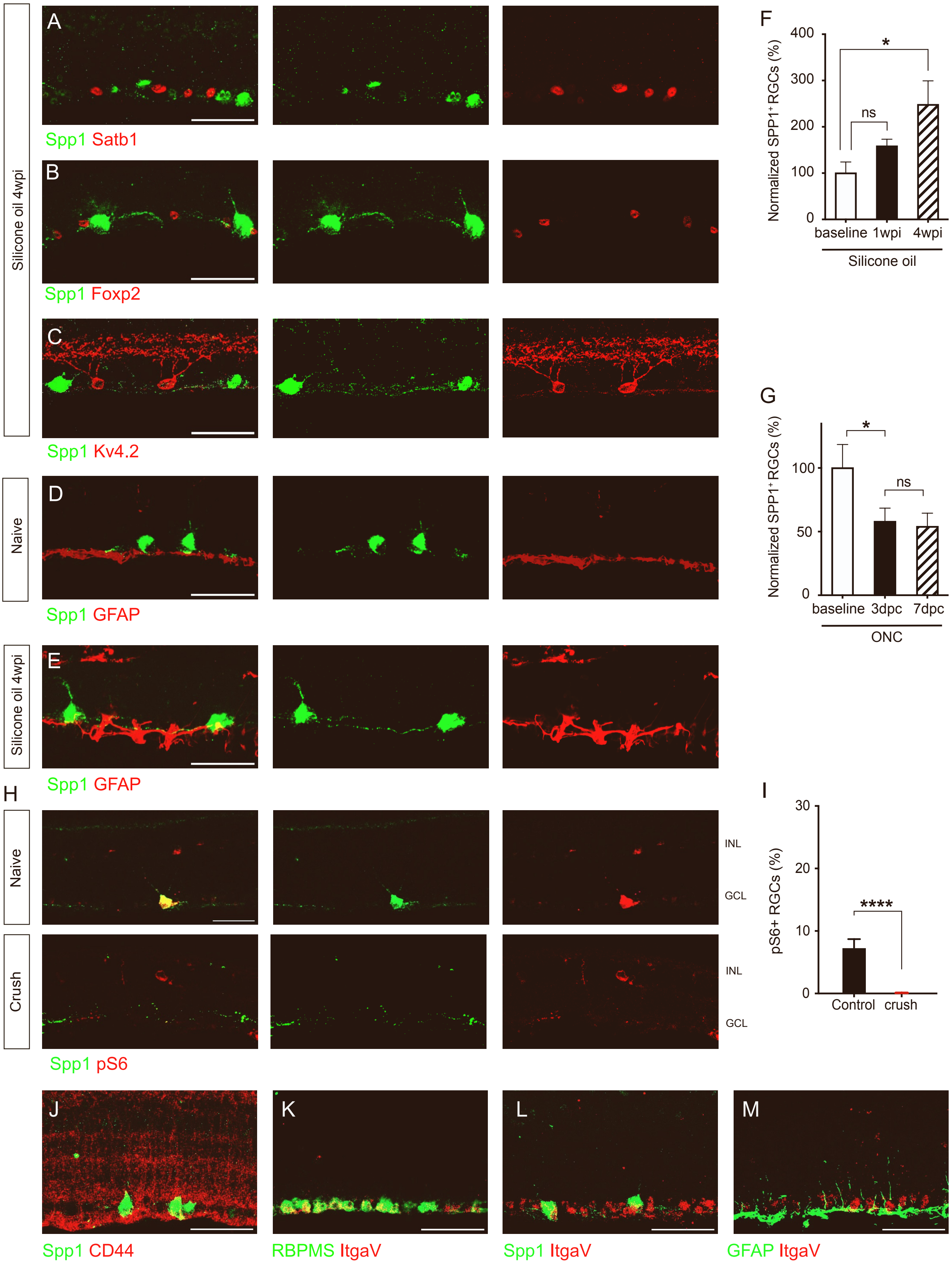

Figure S2 (Related to Figure 2)

Figure S2. Spp1 expression does not increase in ooDSGCs, F-RGCs, Kv4.2-RGCs, or astrocytes subject to SOHU (Related to Figure 2).

(A-C) Sample images for Spp1 (green) and Satb1, Foxp2, and Kv4.2 (red, corresponding to ooDSGCs, F-RGCs, and Kv4.2-RGCs, respectively) showing that few, if any, of these RGC subclasses overlap with silicone oil-induced Spp1 expression. **(D-E)** Sample images for Spp1 (green) and GFAP (red, corresponding to astrocytes) showing that few, if any, astrocytes overlap with Spp1 in either naïve (D) or SOHU conditions (E). Quantifications for (A-E) were shown in Figure 2D, n=5-7 animals per condition. Scale bars, 50μm. **(F)** Quantification of Spp1-expressing RGCs from mice that have undergone silicone oil treatment. Expression is quantified at baseline, 1wpi, and 4wpi. "wpi", weeks post-injection. n=5 animals per condition. One-way ANOVA test. "ns", not significant; "*": $p < 0.05$. **(G)** Quantification of Spp1-expressing RGCs that have undergone ONC treatment at baseline, 3dpc, and 7dpc. "ONC", optic nerve crush; "dpc", days post-crush. n=5 animals per condition. "ns", not significant; "*", $p < 0.05$. **(H)** Vertical section of Kcng4-YFP (αRGCs) naïve retina (top) and ONC at 14dpc (bottom), labeled with antibodies to pS6 and Spp1. Scale bars, 50μm. **(I)** Fractions of the number of RGCs that have high-pS6$^+$ levels in both conditions, while the majority of the pS6$^+$ decrease. n=5 animals per condition. Unpaired t-test; "****", $p < 0.0001$. **(J-M)** Expression of putative receptors for Spp1 in the mouse retina, including CD44 in muller glia (J), as well as ItgaV in RGCs (K)[including αRGCs(L)] but not astrocytes (M). Scale bars, 50μm.

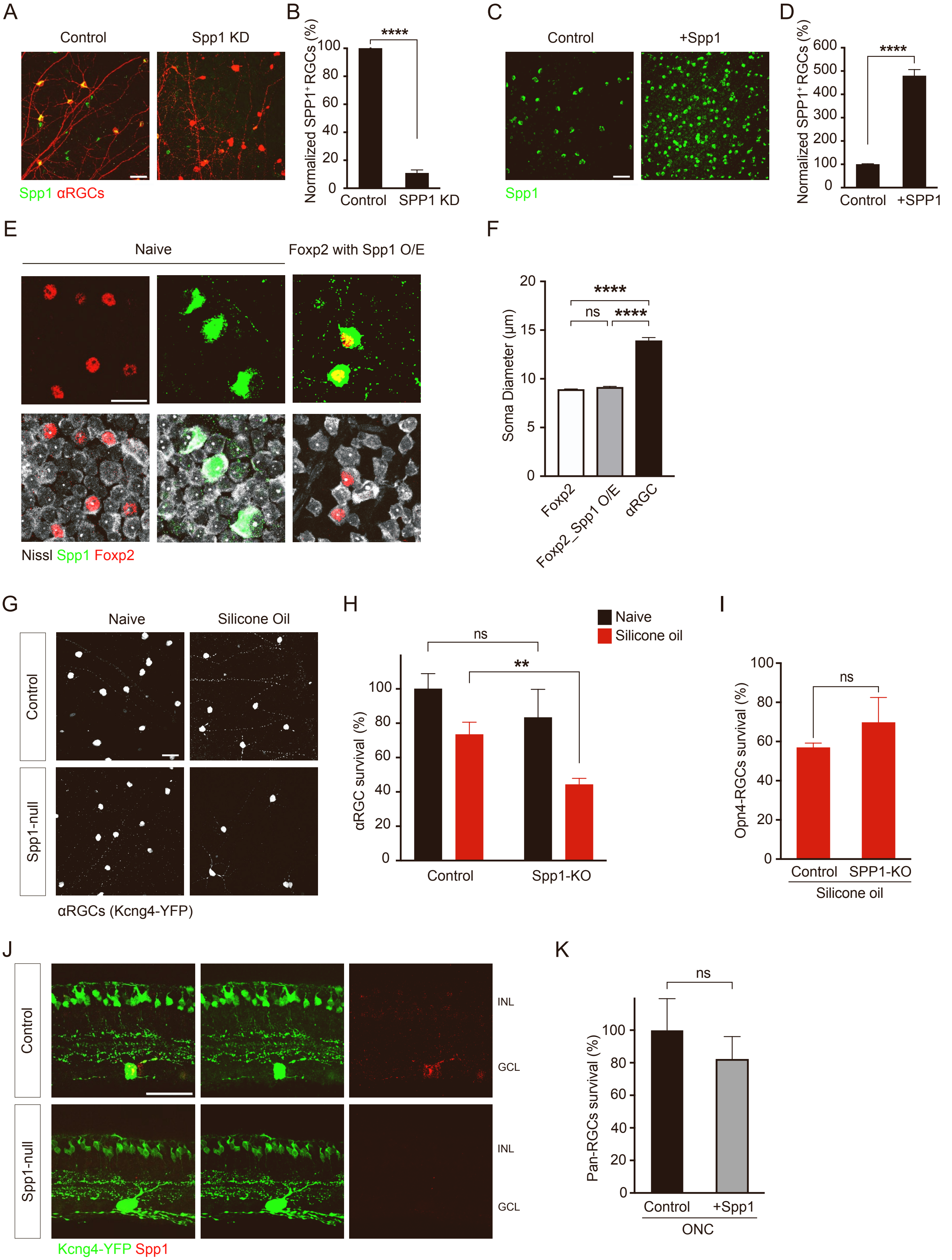

Figure S3 (Related to Figure 3)

Figure S3. Validation of AAV infection for RGC-type specific Spp1 knock-down and overexpression (Related to Figure 3).

(A) Control sgRNA-infected retina (left) and sgSpp1/Cas9 infected retina (right), labeled with antibodies to Spp1 (green) and mCherry (red, highlights AAV infected α-RGCs). **(B)** Quantification of the percentage of Spp1-expressing αRGCs. n=5 retinas per condition. **(C)** Control AAV infected retina (left) and AAV-Spp1 infected retina (right) labeled with antibody to Spp1 in F-RGCs (green). **(D)** Quantification of the percentage of Spp1-expressing F-RGCs. n=5 retinas per condition. **(E-F)** Spp1 overexpression in Foxp2-cre does not change F-RGC properties (E) and does not change their somata size (F). Scale bars, 50µm. n=5 retinas per condition. **(G)** Kcng4-YFP (αRGCs) were compared between control (top) and Spp1 null mutation (bottom), before (left panels) and after silicone oil treatment (right panels). **(H)** Quantification of αRGC survival rate across the four conditions. n=5 retinas per condition. **(I)** Preferential survival of Melanopsin-positive RGCs (M1/M2-RGCs) were not selectively changed in *Spp1* mutants (compared to Figure 1D). n=5 retinas per condition. **(J)** Confirmation of the specificity of Spp1 antibody in *Spp1 mutant* (bottom) compared to controls (top). Scale bars, 50µm. **(K)** Quantification of RGC survival rates between control and Spp1 overexpression at 14dpc, showing no significant Spp1-mediated neuroprotection subject to ONC conditions. Scale bars, 50µm. n=5 retinas per condition. Data are presented as means ± s.e.m in (B, D, F, H, I, K). Unpaired t-test; "ns", not significant; "**", p<0.01; "****", p<0.0001.

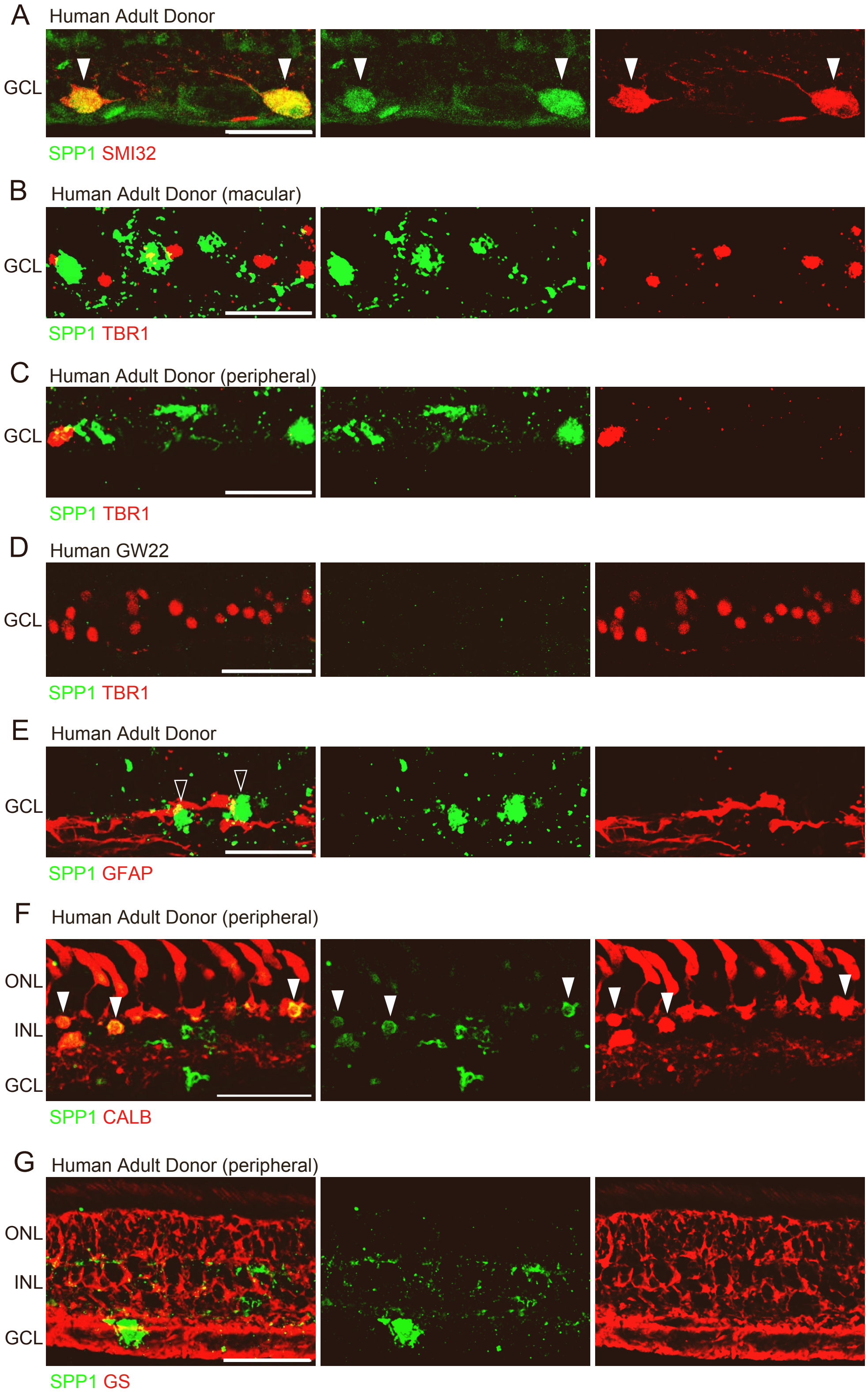

Figure S4 (Related to Figure4)

Figure S4. Data characterizing SPP1-expression in adult human retina and a summary of glaucoma patient information (Related to Figure 4).

(A) Sample images of SPP1 (green) and SMI-32 (red) in the retina from an adult donor sample eye with no notable ocular history. Arrows indicate the overlap of SPP1 and SMI-32. ~18% of SMI-32 was SPP1 positive as an adult human RGC subsets. n= 2 human donor samples. **(B-C)** Sample images of SPP1 (green) and Tbr1 (red) in the macular (B) and peripheral retina(C) from an adult donor sample eye. **(D)** Sample images of SPP1 (green) and Tbr1 (red) in the retina from a GW22 sample eye showing no SPP1 expression at this stage. "G.W.", gestational week. **(E)** Sample images of SPP1 (green) and GFAP (red) in the adult retina showing no SPP1 expression in astrocytes in human retina. Empty arrowheads indicate the SPP1 without GFAP expression . **(F)** Sample images of SPP1 (green) and CALBINDIN (red). Arrows indicate the overlap of SPP1 and CALBINDIN. ~16% of horizontal cells were SPP1-positive in the adult donor sample. **(G)** Sample images of SPP1 (green) and Glutamine Synthetase (GS) (red) indicating SPP1 was not expressed in Muller Glia in the adult donor sample. Scale bars, 50μm.

TABLE S1. Patient demographics along with classification of mild, POAG, severe POAG, and age-matched controls.

		Control	Mild POAG	Severe POAG	p value
**N (%)**		16 (40%)	11 (27.5%)	13 (32.5%)	
**Mean HVF MD**		NA	-2.6 (1.8)	-19.64 (7)	***1.38E-05 (Kruskal-Wallis)
**Mean pre-op IOP (SD)**		16 (2.9)	16.7 (4.2)	20.2 (5.2)	*0.045 (Kruskal-Wallis)
**Mean age (SD)**		75 (7.5)	74.5(4.76)	73.5 (13.92)	0.8649 (Kruskal-Wallis)
**SEX**					0.67 (Fisher's exact)
	Male	6	5	7	
	Female	10	6	6	
**Race**					0.92 (Fisher's exact)
	White	5	5	4	
	black	2	1	3	
	Asian	8	4	4	
	Hispanic	0	0	1	
	Other	1	1	1	
**Laterality**					0.85 (Fisher's exact)
	OD	9	5	7	
	OS	7	6	7	
**Lens status**					0.92 (Fisher's exact)
	phakic	8	5	5	
	pseudophakic	8	6	7	
	aphakic	0	0	1	
**Number of medications**					***9.02E-09 (Fisher's exact)
	0	16	1	0	
	1	0	2	3	
	2	0	3	5	
	3	0	3	3	
	4	0	2	2	
